# Miscellany

**Published:** 1888-07

**Authors:** 


### MISCELLANY.

XANTHELASMA OE THE HEART.— At the VII Congress of Internal Medicine, held at Wiesbaden in April, Leube showed a very rare pathological specimen —a heart affected by xanthelasma. The diagnosis was made during life of mitral insufficiency due to xanthelasma of the endocardium, and the autopsy confirmed the diagonsis.

DANGER FROM THE SANDWICH ISLANDS. —The Consul-General at Honolulu reports that many lepers leave the Sandwich Islands as soon as the disease appears, the greater number coming to the United States, in order to prevent being banished to the island of Molokai.

TREATMENT OF MEDICAL ERYSIPELAS. —For twenty years Jaccoul has used wine of quinquina in large doses (from 250 to 450 grammes a day) and milk diet in facial erysipelas, and has had most satisfactory results from it.—*Journal de Méd. de Paris*, May 20, 1888.

MEDICAL GRADUATES AND THE VIRGINIA EXAMINING BOARD. — It is said that 34 graduates of the College of Physicians and Surgeons of Baltimore have been examined by the Virginia Board of Medical Examiners since January 1, 1885, and that 10 of these failed to pass the examinations.

LITHIATED PILLS FOR DIABETES.—P. Vigier recommends the following formula for pills for diabetes:

Carbonate of lithia........10 centig.
Arseniate of soda ..........3 millig.
Extract of gentian.........5 centig.

For one pill. Take one morning and evening, and continue after the sugar has disappeared from the urine.

WASH FOR SYPHILITIC PSORIASIS. —

Bichloride of mercury.......1 gram me.

Muriate of ammonia........1 "

Dissolve in two litres of tepid water, and wash the affected parts, in cases of plantar and palmar syphilitic psoriasis, night and morning.—*L'Union Médicale*, May 26, 1888.

A monument to Cohnheim was unveiled in Leipzig on June 3.

